# Effect of the Load Application Angle on the Compressive Behavior of Al Honeycomb under Combined Normal–Shear Stress

**DOI:** 10.3390/ma16155462

**Published:** 2023-08-04

**Authors:** Giulia Arquilla, Alessandra Ceci, Girolamo Costanza, Maria Elisa Tata

**Affiliations:** Industrial Engineering Department, University of Rome Tor Vergata, Via del Politecnico 1, 00113 Rome, Italy; giulia.arqui@gmail.com (G.A.); alessandraceci1996@gmail.com (A.C.); elisa.tata@uniroma2.it (M.E.T.)

**Keywords:** mechanical characterization, Al honeycomb, combined normal–shear stress, compressive behavior

## Abstract

A comparison of the compressive behavior of Al honeycomb under pure normal stress and combined normal–shear stress was analyzed in this work. The typical working stress of honeycomb is a compressive load along the direction parallel to the axis of the cells. However, the component can also undergo shear stresses during operation, which can cause premature failure. This work analyzes the mechanical behavior in compression by normal stress (0°) and in conditions of combined normal–shear stress (at 15° and 25°) using a special pair of wedges. The samples were obtained from a 3000 series Al alloy sandwich panel and tested according to the ASTM C365/C365M-22 standard. The different deformation modes of the cells in the combined compression were examined for three angles (0, 15°, and 25°). A theoretical model of combined compression was used to derive the normal and tangential components starting from the total stress–strain curves. A compression curve analysis was conducted at different angles θ, allowing for considerations regarding changes in strength, absorbed energy, and deformations. Overall, as the load application angle increased, both the shear resistance of the honeycomb and its tangential displacement up to densification increased, which is the opposite of what occurs in normal behavior. The cell rotation angle was calculated as the load angle varied. The rotation angle of the cell increased with the displacement of the crosshead and the application angle of the force.

## 1. Introduction

In recent years, composite sandwich structures have been widely used as structural elements [1], especially in the aerospace field [2], naval sector [3], and construction of high-performance vehicles [4]. The main purpose is to obtain structural components characterized by a high bending stiffness and, meanwhile, a reduced weight compared to traditional materials [5]. A sandwich structure is made up of a central structure called the ‘core’ and two external sheets called the ‘skins’. These skins are usually composed of fiber-reinforced laminates and are responsible for the mechanical properties of the materials. The core is made of a low-density material, such to allow lightness of the structure, and it has the main task of assembling the skins and transfer the loads to them. The possibility of employing a low-density material allows the use of a higher thickness for the internal part by increasing the inertia momentum of the cross-section and reducing the stress of the external part of the section: a wide range of constructive solutions are available, and the choice of materials adopted for the construction of sandwich structures [6,7]. Some advantages of sandwich constructions are the following: high strength in crash and impact [8,9,10], thermal and acoustic insulation [11], good resistance to chemical agents, and suitability for food contact.

**Figure 1:**
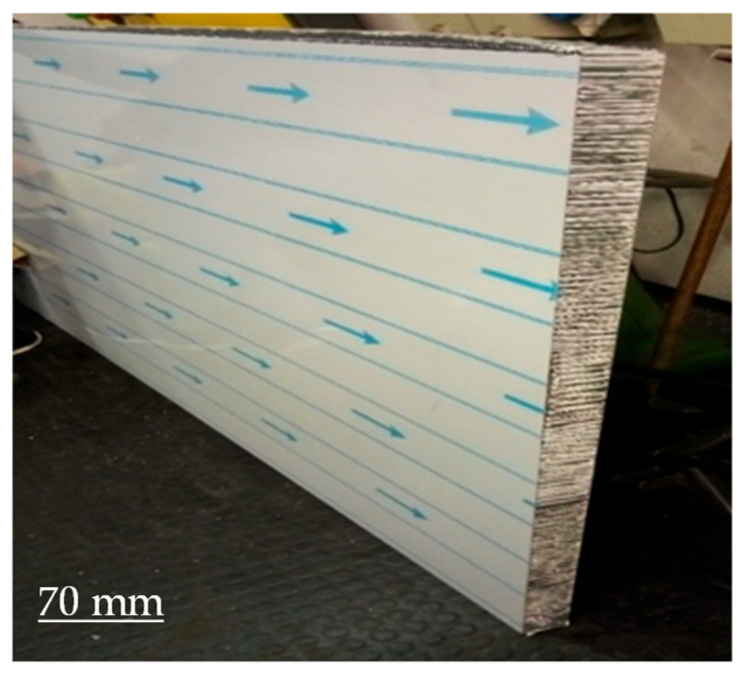
Sandwich structure with honeycomb core.

The most common material employed for the core is honeycomb. Such a structure derives its name from its beehive shape, which is made up of many cells that can have different shapes and sizes. Honeycomb structures, with density between 20 and 200 kg/m^3^, are nowadays reproduced with many materials, reinforced and not-reinforced polymers, metals, and alloys. Aluminum is the material most used in metal honeycomb due to its low density. To obtain the maximum stiffness and bending strength, it is necessary that the weight of the honeycomb is in the range of 50–66.7% of the weight of the skin panels of the sandwich structure. In Figure 1, a sandwich structure with a honeycomb core is reported.

**Figure 2:**
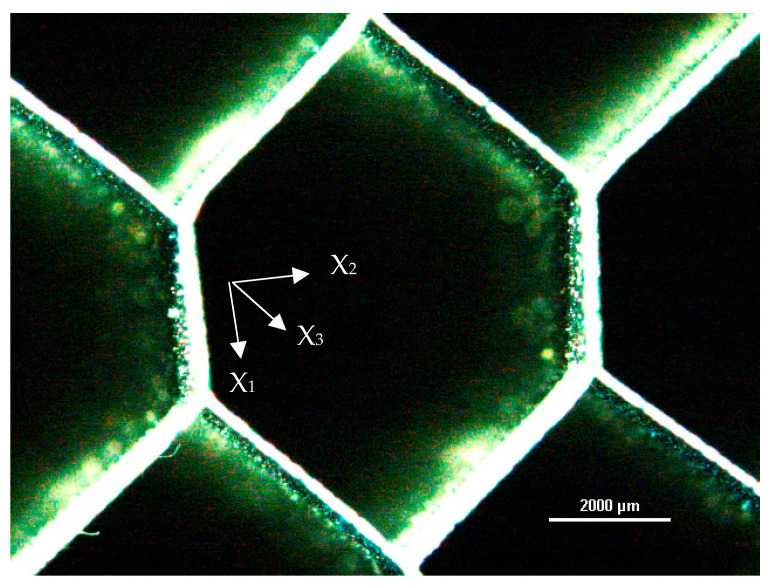
Base cell geometry.

The compressive behavior of metal honeycomb is strongly influenced by the geometry of the cell (“*t*” cell wall thickness and “*l*” cell wall length), as shown in Figure 2, by the properties of the cellular wall material and, in particular, by the relative density, expressed as in (1) in the hypothesis that *t*/*l* (and consequently ρ*/*ρ_s_*) are small and take into account the double thickness due to the manufacturing process:

(1) $$\frac{\rho^{*}}{\rho_{S}}=\frac{\left(\frac{t}{l}\right)\left(\frac{h}{l}+1\right)}{cos(\theta )(\frac{h}{l}+sin\theta )}$$

For a regular hexagonal cell, the relationship is valid [5]:

(2) $$\frac{\rho^{*}}{\rho_{s}}=\frac{8}{3\sqrt{3}}\frac{t}{l}=\frac{8}{3}\frac{t}{S}$$

The typical cell structure makes honeycomb, and, consequently, honeycomb-based sandwich panels, intrinsically anisotropic and, in detail, orthotropic, that is, with three elastic symmetrical axes orthogonal between them. Considering the reference system, in which the axes are parallel to the straight line generated by the intersection of such planes, as with X_1_–X_2_–X_3_ in Figure 2, in this way, nine independent elastic constants can be obtained [5]. In terms of the out-of-the-plane compression, this is usually called compression out-of-the-plane when the honeycomb is subjected to a state of compressive uniaxial tension in the direction parallel to the longitudinal cell length. Under such stress, the honeycomb structure results in being much stiffer. The elastic modulus $E_{3}^{*}$ is that of the wall material divided by the relative density [5], as reported in Equation (3).

(3) $$E_{3}^{*}=\frac{E_{s}\rho^{*}}{\rho_{s}}$$

The inverse constitutive relationship, $\varepsilon_{ij}=C_{ijkl}\sigma_{kl}$, thus becomes:

(4) $$\left[\begin{array}{c} \varepsilon_{11}^{*}\\ \varepsilon_{22}^{*}\\ \varepsilon_{33}^{*}\\ \gamma_{23}^{*}\\ \gamma_{13}^{*}\\ \gamma_{12}^{*} \end{array}\right]=\left[\begin{array}{cccccc} \frac{1}{E_{1}^{*}} & -\frac{\nu_{21}^{*}}{E_{2}^{*}} & -\frac{\nu_{31}^{*}}{E_{3}^{*}} & 0 & 0 & 0\\ -\frac{\nu_{12}^{*}}{E_{1}^{*}} & \frac{1}{E_{2}^{*}} & -\frac{\nu_{32}^{*}}{E_{3}^{*}} & 0 & 0 & 0\\ -\frac{\nu_{13}^{*}}{E_{1}^{*}} & -\frac{\nu_{23}^{*}}{E_{2}^{*}} & \frac{1}{E_{3}^{*}} & 0 & 0 & 0\\ 0 & 0 & 0 & \frac{1}{G_{23}^{*}} & 0 & 0\\ 0 & 0 & 0 & 0 & \frac{1}{G_{13}^{*}} & 0\\ 0 & 0 & 0 & 0 & 0 & \frac{1}{G_{12}^{*}} \end{array}\right]\left[\begin{array}{c} \sigma_{11}^{*}\\ \sigma_{22}^{*}\\ \sigma_{33}^{*}\\ \tau_{23}^{*}\\ \tau_{13}^{*}\\ \tau_{12}^{*} \end{array}\right]$$

**Figure 3:**
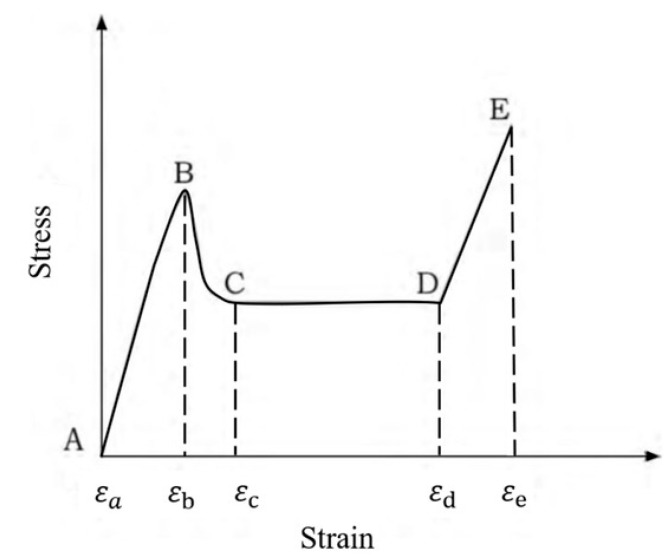
Typical stress–strain curve for compression out-of-the-plane of metallic honeycomb [12].

The elastic modulus $E_{3}^{*}$ is of the base material and is reduced by the relative density [5]. A typical stress–strain curve is shown in Figure 3.

The curve reported in Figure 3 shows the typical steps of a compressive curve: the initial pseudo-elastic stage up to the maximum pick stress. After the peak, the stress reaches a local minimum due to the presence of the first instability folds in the plastic regime [10]. After that, a huge plateau region in which numerous undulations are visible, characteristic of the genesis of successive instability lobes on the cell walls, with high energy absorption, is identified. This is in good agreement with the applications of such structures, in which excellent energy absorption and impact resistance are required. Finally, the densification stage is present, in which a steep stress increase is evident due to the final collapse of the cell walls, and no more deformation can occur. Characteristic values of the curve, such as $\sigma_{pk,3}^{*}$, $\sigma_{m,3}^{*}$, $\varepsilon_{d}^{*}$, $and\;U^{*},$ have been obtained from many models available in the literature [5].

## 2. Motivation and Novelties

The typical loading condition of honeycomb and sandwich structures is compression in the direction parallel to the cell axis. A shear stress component may be applied during operation, which could cause a premature failure of the component. Furthermore, in a real crash event, the impact loading is much more complex than pure compression. The loading condition is rarely uniaxial. A combination of compression and shear is generally more plausible. In the past, complex solutions and configurations have been introduced as biaxial or multiaxial testing machines, and three load cell machines or double specimens are symmetrically placed and simultaneously crushed to eliminate transverse loads and protect the machine. The motivation at the basis of this work is the experimental activity, which aims at highlighting the differences between stress with a normal load (0°) and stress with a combined normal–shear load, by the means of wedges inclined at 15° and 25° with a standard tensile machine. The loading conditions were operated with the aid of a pair of wooden wedges, purposely sized and shaped to be installed, with appropriate special supports, on the compression machine. The first phase of the experiment allowed for the sizing and construction of the wedges with the relative anchor planes to the machine. The additional tools were suitably studied and created only after preliminary tests were carried out on the compression machine. After that, the specimens were prepared in size and shape, as described in the ASTM C365/C365M-22 standard [13], from a commercial sandwich panel in a 3000 series Al alloy. The second phase of the experimental campaign involved the execution of compression tests carried out at room temperature. This allowed for the extrapolation of the stress–strain curves at 0, 15°, and 25° load application angles on different specimens. The theoretical model of the combined compression was derived from an analysis in the literature [12,14,15,16], based on the forces decomposition in different reference systems, one relative to the crosshead and the other relative to the wedges. In the cited works, similar experiments were carried out on aluminum honeycombs at different load angles θ, with different test apparatus. An earlier work [17] of some of the authors was the starting point; however, the forces acting on the surface of the specimen were the same and the relationship between them was described from the empirical relationship already obtained. From the experimental tests, it was possible to obtain the total stress–strain curves. Successively, each curve was decomposed into the relative components of normal and shear. A morphological analysis was carried out on the morphology of the post-compression specimens and the graphs were compared with the variation in the θ angle, allowing for some considerations to be made regarding the variation in the strength, in the absorbed energy, and in the deformations that occurred. Finally, the cell rotation angle was calculated as the load angle varied.

## 3. Materials and Methods

### 3.1. Samples Preparation and Geometrical Characterization

**Figure 4:**
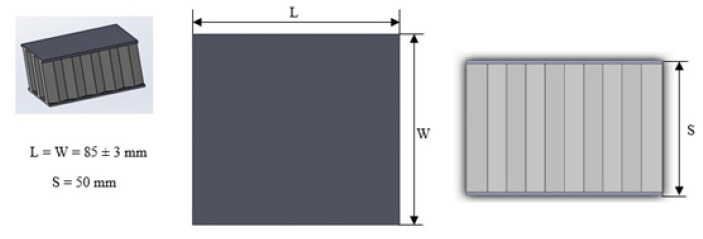
Sandwich sample dimensions.

**Figure 5:**
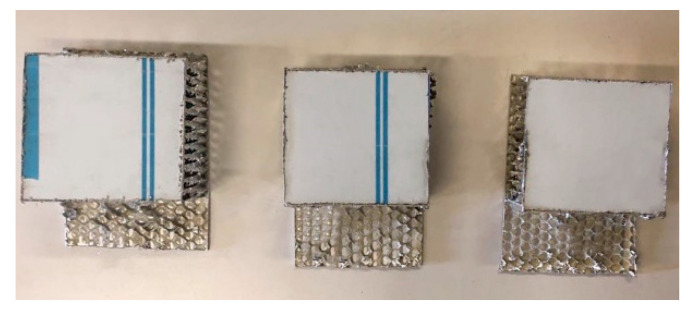
Sandwich samples ready for compression tests.

In order to perform the static compression tests, honeycomb samples were extracted from a large-size panel by the means of a miter saw with a special alumina cutting disc. In this way, samples with a square cross-section with a 85 +/− 3 mm side and standard thickness of the panel (50 mm) were obtained. A sketch of the manufactured samples is reported in Figure 4 and pictures are shown in Figure 5.

**Figure 6:**
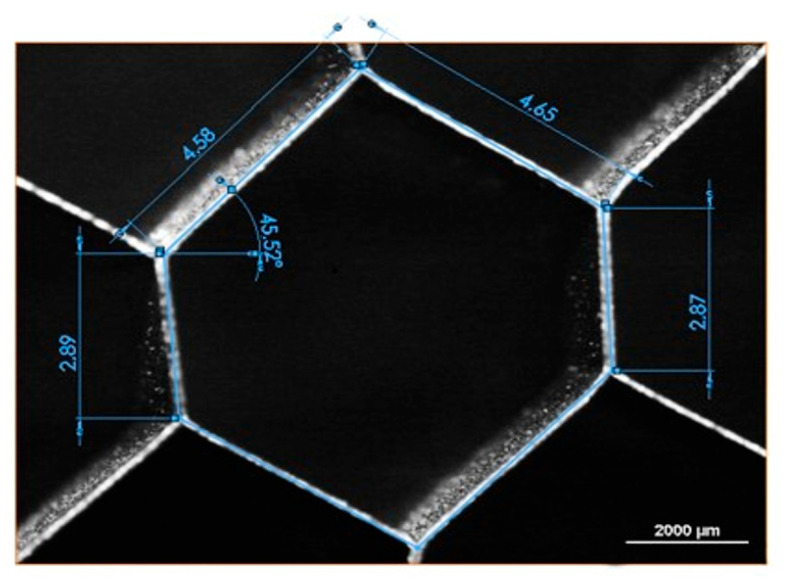
Geometric features of the hexagonal honeycomb cells: l = 4.6 mm, h = 2.9 mm, and q = 45°.

**Figure 7:**
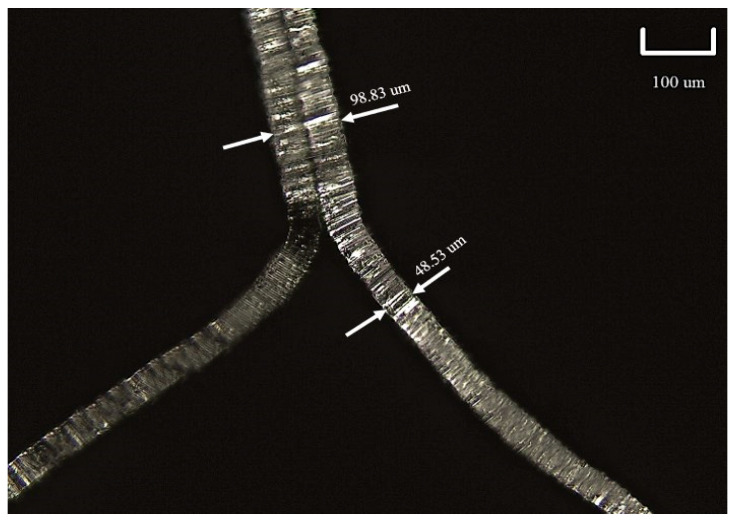
Optical microscope micrograph with wall thickness measurements.

The base geometry of the hexagonal honeycomb was analyzed through observations at the stereo-microscope of a small number of cells, which exhibited minimum internal defects. In Figure 6, the average geometric values taken from the analysis and a micrograph of the observed cell are reported. The wall thickness was measured through a micrograph taken from the optical microscope: the average thickness was about 0.05 mm, as evidenced in Figure 7.

In Figure 7, it is possible to find the round geometric defect on the corner of the node and the double thickness walls due to the manufacturing process. With such data, the relative density can be estimated at 0.019, without considering the contribution due to the imperfection illustrated above.

### 3.2. Experimental Apparatus Setup

**Figure 8:**
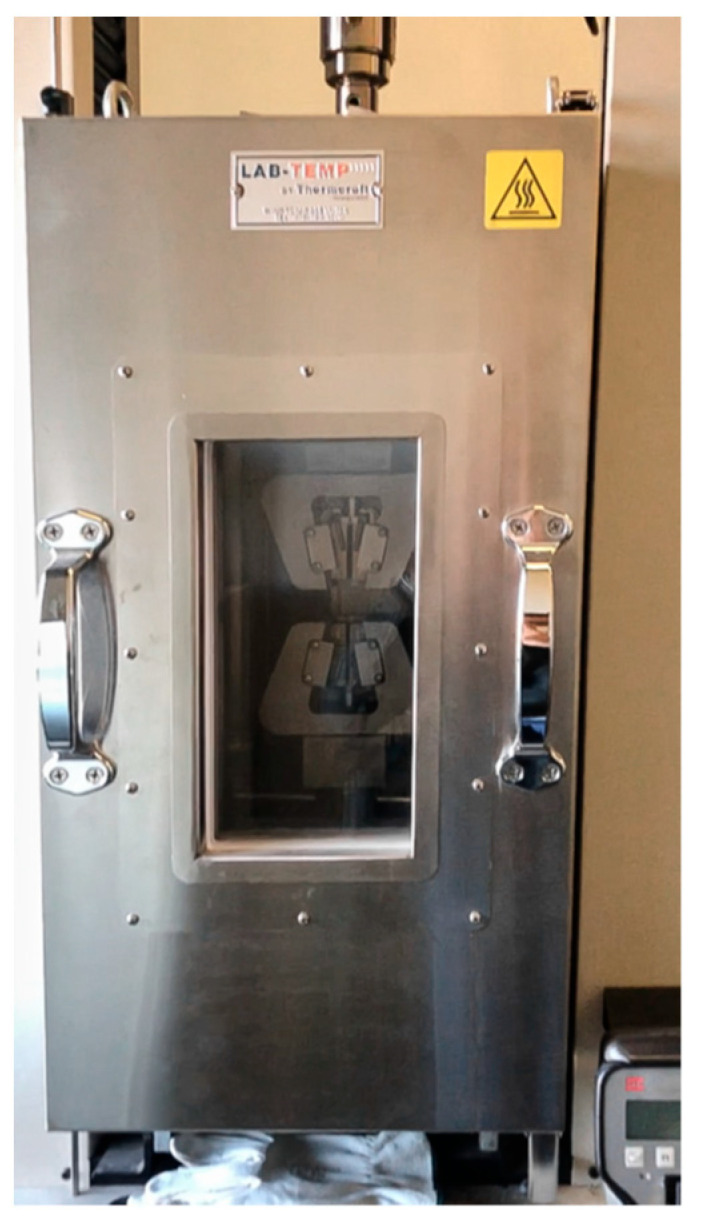
MTS Insight 50 tensile machine with thermostatic room.

**Figure 9:**
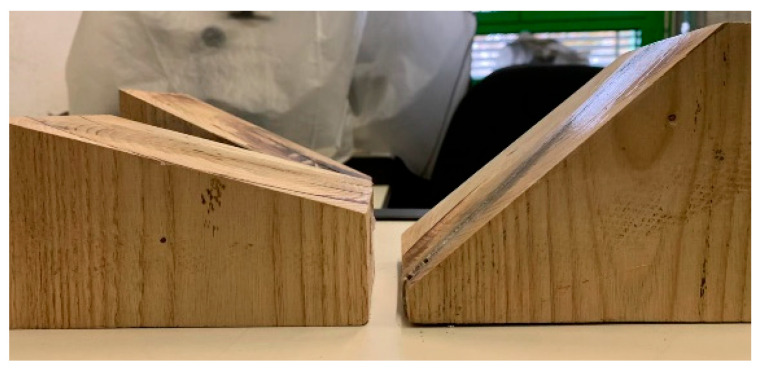
Wooden wedges with different angles.

The compression tests were performed using the MTS Insight 50 machine, employed to perform the tests at 0°, a configuration in which the sample was subjected only to pure normal stress (Figure 8). In order to characterize the honeycomb samples in conditions of combined normal + shear stress, different couples of wooden wedges with different angles were designed and built up (Figure 9). The shape and size of such wedges were tailored so that the samples to be compressed could be easily mounted on the machine and tested.

**Figure 10:**
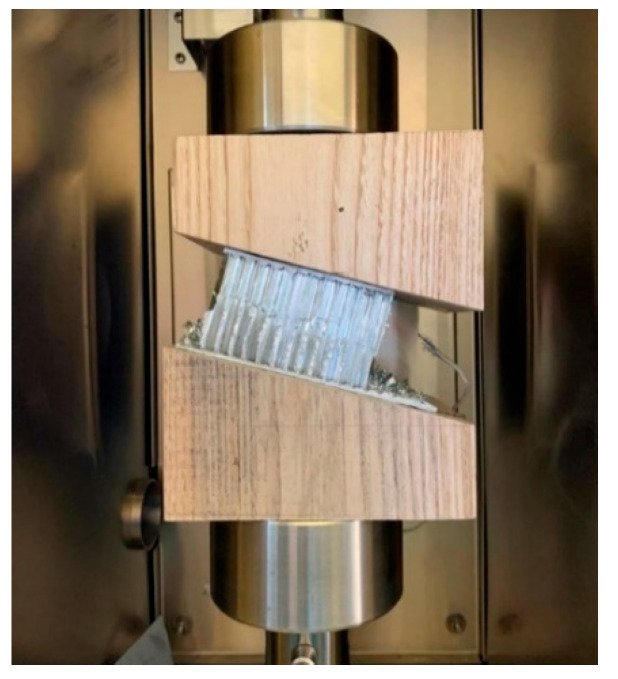
Preliminary tests for the definition of the locking system and the lateral constraint.

In Figure 10, a honeycomb sample mounted on a couple of 15° wedges is shown at the beginning of the compression test, without any lateral constraint.

**Figure 11:**
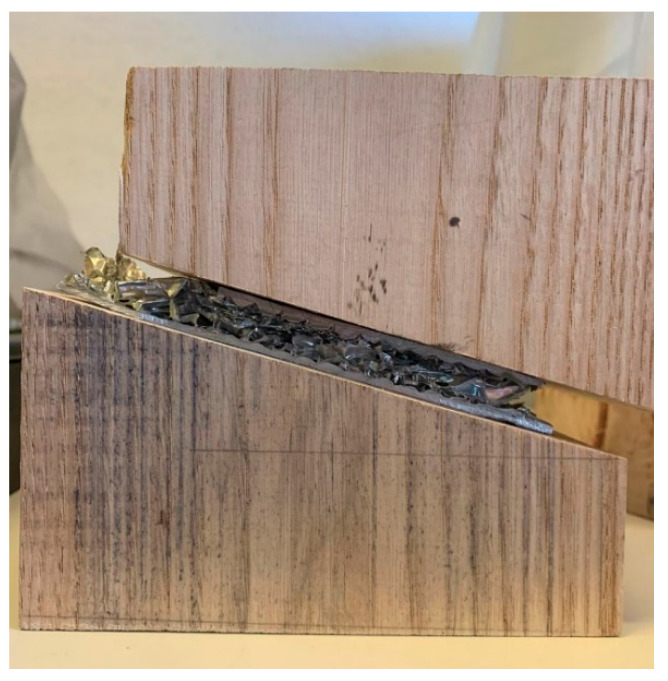
Lateral shift of the wedges during compression tests without any lateral constraint.

In Figure 11, a lateral shift of the wedges is evidenced at increasing successive deformation steps, without any lateral constraint of the machine.

**Figure 12:**
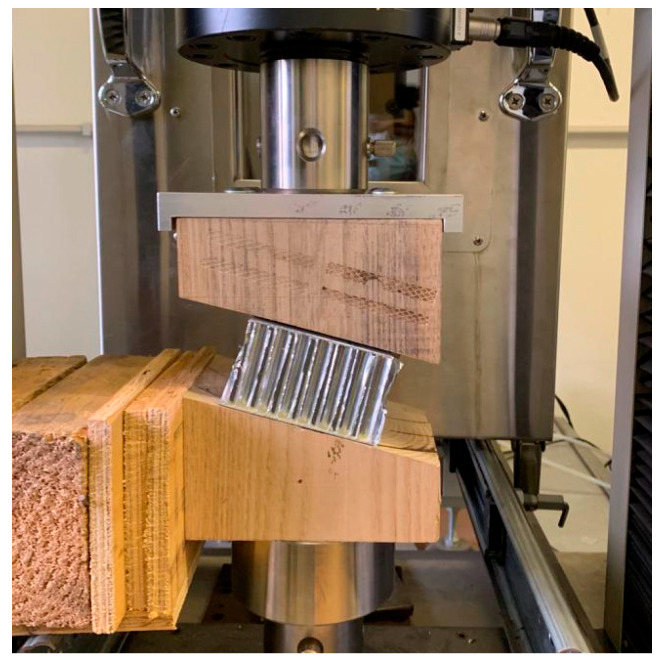
Anchoring system and supports for the wedges of the MTS Insight 50 tensile machine.

To obtain reliable results from the compression tests, considering that higher angles imply a greater shear stress on the tensile machine, the upper wedge was fixed to a platen by the means of inox screws applied to the wood. This device allowed a wedge to be fixed on the crosshead of the tensile machine, while the lower wedge was left free vertically, with specific lateral supports in order to avoid the lateral shift, as shown in Figure 12.

### 3.3. Validation of the Test Apparatus

**Figure 13:**
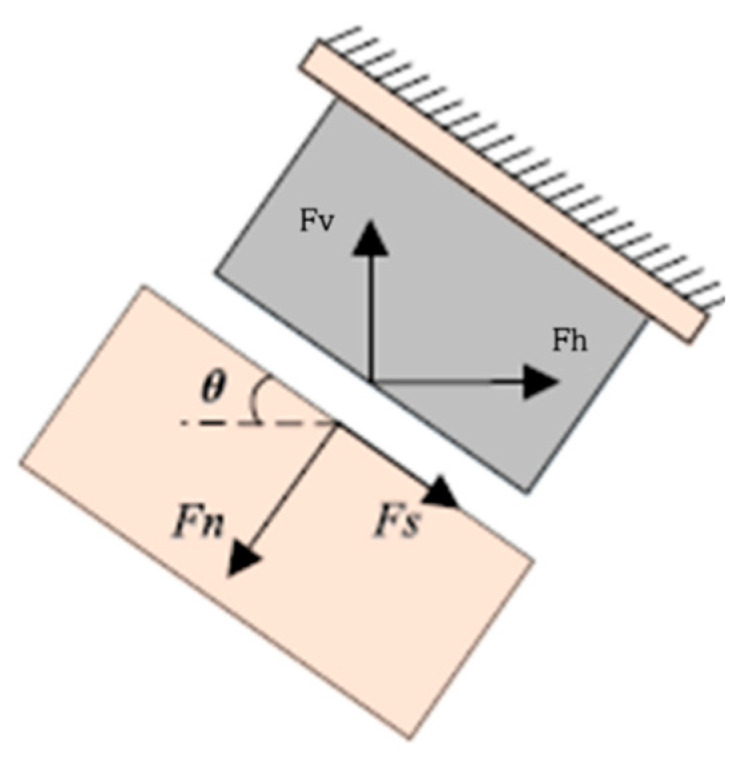
Forces acting on the machine and on the sample [15].

In the configuration of the combined normal–shear stress, the forces acting on the sample can be discomposed in the component acting horizontally and vertically on the machine (*F_v_* and *F_h_*, respectively), while the normal and tangential forces acting on the samples are *F_n_* and *F_s_*, respectively. *θ* is the angle of application force and the relationships between the various forces are reported in the following Equations (5) and (6), as evidenced in Figure 13:

(5) $$F_{h}=F_{n}sin\theta -F_{s}cos\theta$$

(6) $$F_{v}=F_{n}cos\theta +F_{s}sin\theta$$

**Figure 14:**
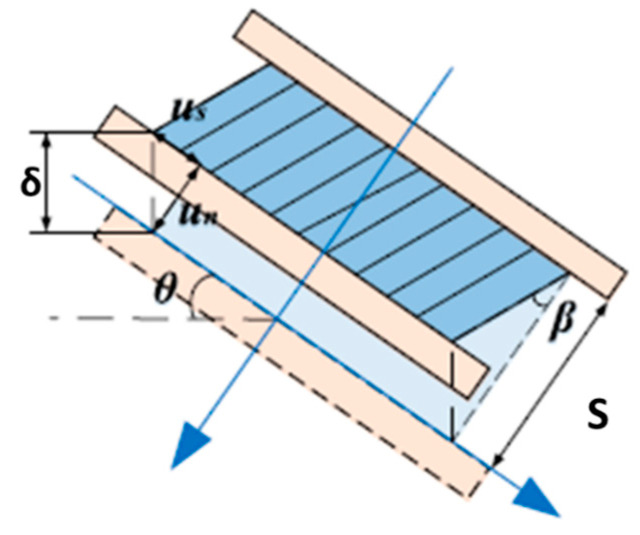
Rotation of the cell axis and displacement along the normal and tangential directions [15].

Considering the rotation of the axis of the honeycomb cell, under a load angle greater than 0°, as shown in Figure 14, assuming an infinite friction coefficient between the honeycomb sample and the wedge placed on the crosshead machine under the hypothesis of normal and uniform shear stress, the relationship between the angular rotation of the cell’s axis and the displacement in the normal and tangential directions can be expressed as reported in the following Equations (7)–(9):

(7) $$u_{n}=\delta cos\theta$$

(8) $$u_{s}=\delta sin\theta$$

(9) $$tan\beta =\frac{u_{s}}{S-u_{n}}=\frac{u_{n}tan\theta }{S-u_{n}}=\frac{\varepsilon^{*,n}tan\theta }{1-\varepsilon^{*,n}}$$

where *β* is the rotation angle of the cell axis; *δ*, *u_s_*, and *u_n_* are, respectively, the vertical displacement, the normal displacement, and the shear displacement of the load application system; and $\varepsilon^{*,n}=\frac{u_{n}}{S}\;$ is the normal strain of the sample. It can be seen that the rotation angle depends only on the starting load angle and the normal strain of the sample.

According to the test standard ASTM C365/365M-22 for determining the values of the stress and strain, both for the pure compression and combined normal–shear compression, the following can be referred to:

(10) $$\sigma^{*,v}=\frac{F_{v}}{A_{p}}$$

(11) $$\epsilon^{*,v}=\frac{\delta }{S}$$

While normal and shear stress can be defined as:

(12) $$\sigma^{*,n}=\frac{F_{n}}{A_{p}}$$

(13) $$\tau^{*}=\frac{F_{s}}{A_{p}}$$

where *A_p_* is the cross-sectional area.

## 4. Experimental Results

The compression tests were carried out at different application force angles (0, 15°, and 25°). For each loading condition, two samples were tested in order to check the repeatability of the process. The stress reported in the next graph was calculated using Equation (10), that is, the vertical stress corresponding to the vertical force *Fv*, measured by the load cell of the tensile machine. The corresponding deformation in the vertical direction is computed through Equation (11). The samples were characterized, while the data were declared by the supplier and are reported in Table 1 [18].

At the end of the experimental tests, the following engineering properties (reported in Table 2) were obtained.

For each test, the “toe region” was compensated for, a zone of the stress–strain curve that does not reflect real material properties, but can be attributed to a moderate misalignment of the test sample. After this correction, the right positioning on the x-axis was achieved. This correction was repeated for all the tests performed with increasing loading application angles.

### 4.1. Normal Compression Tests (0°)

**Figure 15:**
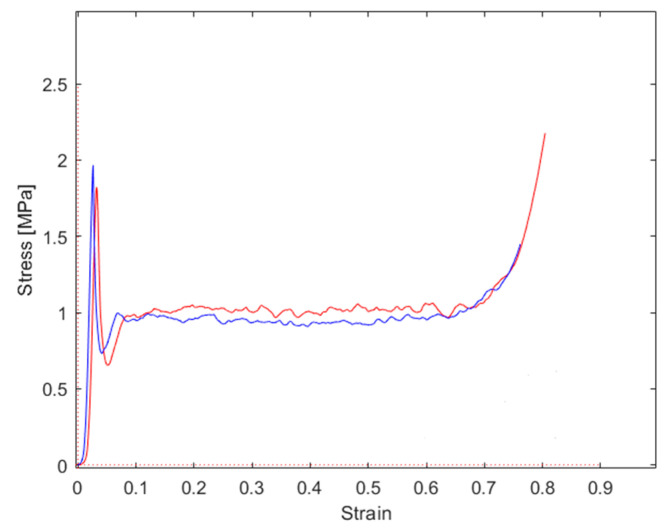
Stress–strain graphs for repeated tests, both performed at 0° load application angle.

The stress–strain graphs for the tests performed at a 0° force application angle are reported in Figure 15; the main results are summarized in Table 3. As evidenced from the graph, a good repeatability of the properties was evidenced. Both the analyzed samples (tests were presented twice in the same loading conditions to check the repeatability) showed a peak stress in correspondence with the instability of the cell walls, followed by a sharp stress decrease and successively by the huge plateau region in which the stress was nearly constant. After that, the densification appeared. The peak stress was nearly twice the plateau stress. The final densification occurred at about 80% of the strain.

### 4.2. Combined Normal–Shear Tests (15°)

**Figure 16:**
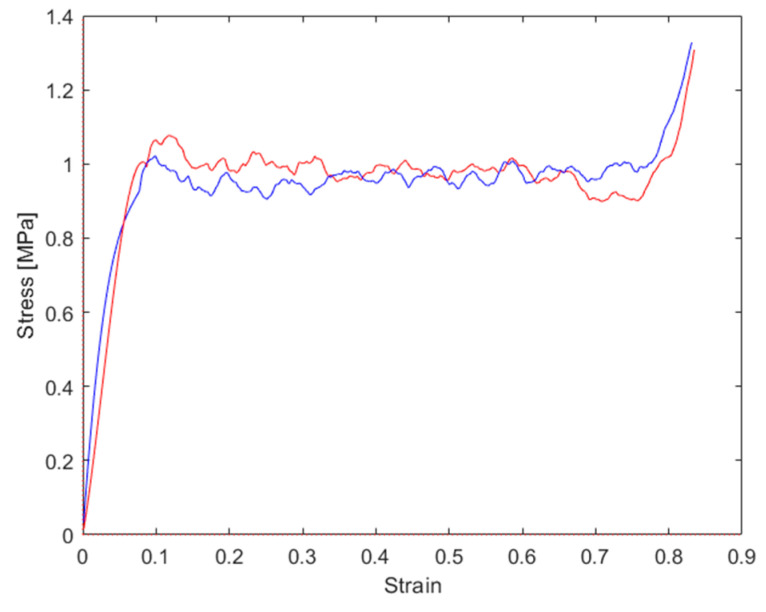
Stress–strain graphs for repeated tests, both performed at 15° load application angle.

The stress–strain graphs for the tests performed at a 15° force application angle are reported in Figure 16 and the main results are summarized in Table 4. Compared to the tests performed at a 0° application angle, the stress dropped due to the instability no longer being present as a consequence of the shear component acting on the honeycomb cells. The peak stress was moderately higher than the plateau stress. The final densification occurred at 80% of the strain.

### 4.3. Combined Normal–Shear Tests (25°)

**Figure 17:**
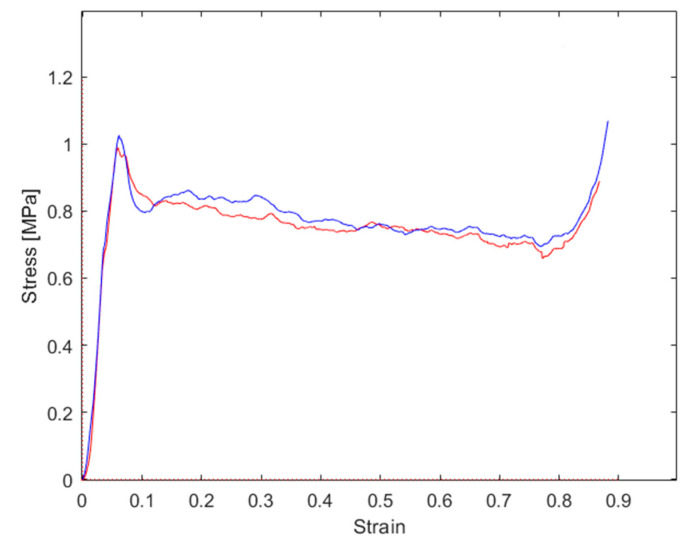
Stress–strain graphs for repeated tests, both performed at 25° load application angle.

The stress–strain graphs for tests performed at a 25° force application angle are reported in Figure 17 and the main results are reported in Table 5. Compared to the tests performed at a 0° application angle, the stress dropped due to the instability no longer being present as a consequence of the shear component acting on the honeycomb cells. The peak stress was moderately higher than the plateau stress. The plateau in this load condition exhibited a moderate decrease in stress. The final densification occurred at about 85% of the strain.

## 5. Results Analysis

### 5.1. Stress–Strain Curves at Different Load Application Angles

**Figure 18:**
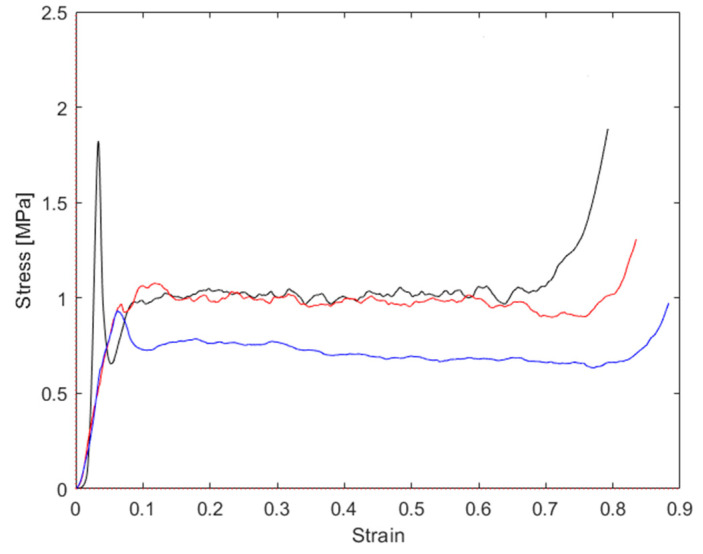
Total stress–strain graphs for samples compressed at 0° (black curve), 15° (red curve), and 25° (blue curve).

**Figure 19:**
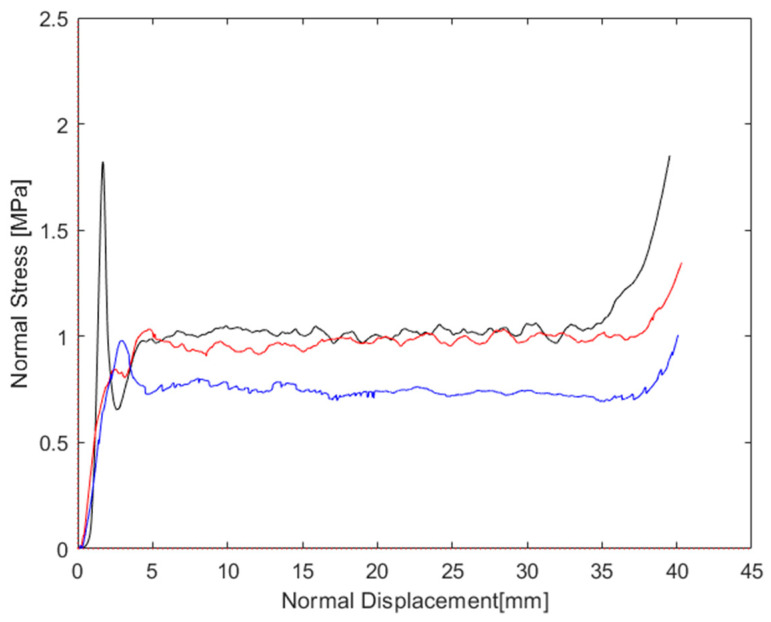
Normal stress–displacement graphs for samples compressed at 0° (black curve), 15° (red curve), and 25° (blue curve).

**Figure 20:**
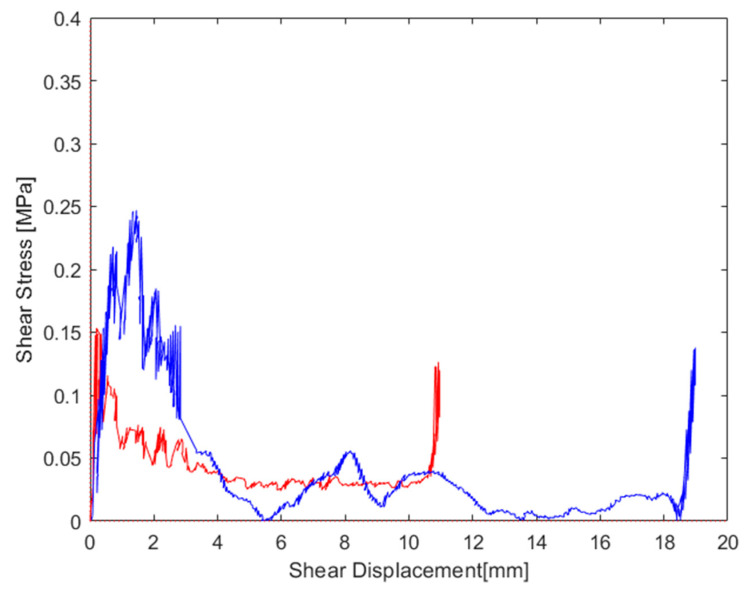
Shear stress–displacement graphs for samples compressed at 15° (red curve) and 25° (blue curve).

The graphs reported in Figure 15, Figure 16 and Figure 17 were derived from the output data of the compression machine, calculated from all the stress–strain curves. For an immediate comparison, such results are summarized in Table 6. A deeper analysis based on the decomposition of the normal stress and shear stress, with each of them plotted vs. normal strain and shear strain, respectively, allows for the finding and separating of each contribution. In Figure 18, the curves the total stress total strain are shown. Starting from Equations (7) and (8), the normal and shear displacement ($u_{n}$,$u_{s})$, reported in the x-axis of Figure 19 and Figure 20, were derived from the total vertical displacement δ. On the Y-axis of Figure 19 and Figure 20, the values of the normal and tangential stresses are reported.

In Equation (6), the vertical force *Fv*, normal force *Fn*, and *Fs* are correlated. This relationship was obtained from the analysis of the many curves in the literature [14,15,16], based on the section and height of the specimens. From the knowledge of such values, it is possible to associate with each known force value (*Fv*) a corresponding component of the shear component *Fs*. Such a relationship, *Fs* = *f*(*Fv*, *d*, *q*) was used to obtain the *Fs*. At the end, the known *Fv*, *Fs*, and *θ*, using Equation (6), found the values of *Fn*. In Figure 19 and Figure 20, the curves of the normal-stress–normal-displacement and shear-stress–shear-displacement are reported.

**Figure 21:**
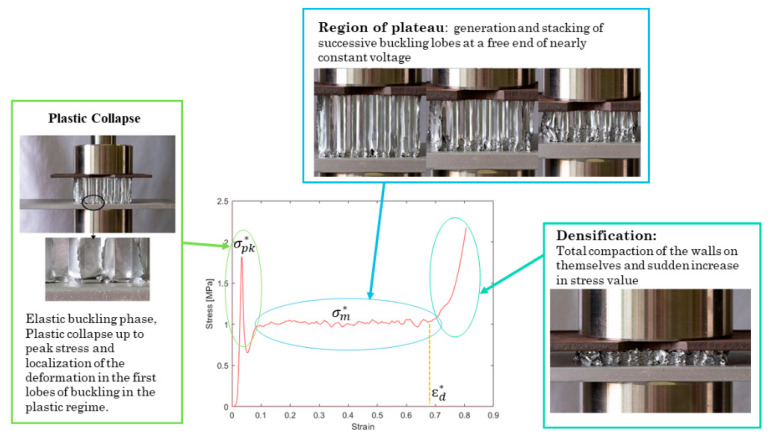
Steps of compression out-of-the-plane for honeycomb and relationship with the compression curve.

The main steps of the compression out-of-the-plane for honeycomb are reported in Figure 21, where in each zone of the curve $\sigma^{*}-\epsilon^{*}$, after the initial pseudo-elastic stage, the corresponding pictures during the compression are associated. As mentioned above the combined compression–shear load was applied at different angles (0°, 15°, and 25°) for the honeycomb under investigation. All the samples were fully compressed until final densification (i.e., when the load increased sharply). In all three load application angles, the tested honeycombs exhibited three deformation stages (Figure 21):

(1) The pseudoelastic phase, in which the stress linearly increased with the strain; this stage was characterized by elastic instability in the samples loaded at 0° and plastic collapse up to peak stress;
(2) The plateau phase, in which the stress was nearly constant (for 0° and 15°) and moderately decreasing (for 25°);
(3) The densification phase, in which the stress showed a sharp increase due to the end of the plasticity of the honeycomb cell walls.

The effects of the force application angle on the crushing stress are evident from Figure 18, where it can be observed that, for an angle of 15°, the stress in the plateau phase was almost constant, while, for an angle of 25°, the stress in the plateau phase slightly decreased with the strain. Since a decreasing trend of the stress was already present in the curve at 15°, while it was much more evident with an angle of 25°, it is hypothesized that the decreasing trend of stress seemed to become more significant as the load angle increased.

### 5.2. Cell Rotation Angle

**Figure 22:**
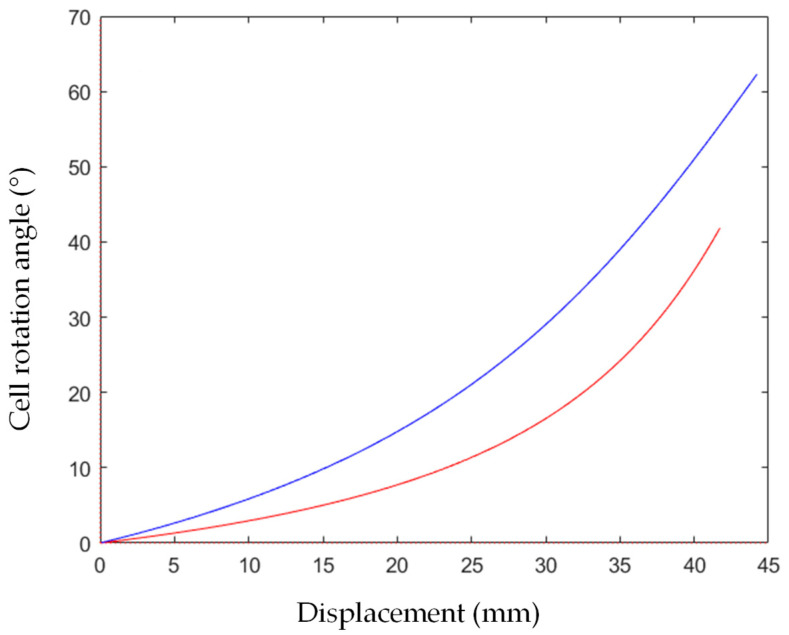
Cell rotation angle for 15° (red curve) and 25° (blue curve) load application angle.

As mentioned in the previous paragraph, the rotation angle of the cell increased with the displacement of the crosshead (and consequently with the strain) and the force’s application angle, as described in Equation (9) and schematized in Figure 14. The rotation angle was calculated for the various angles of application of force and is reported in Figure 22. It can be noted that the increase in the rotation angle was more evident at 25° in comparison to that obtained at 15°.

## 6. Discussion

Comparing the compression curves, it was evident that the shear stress and normal stress under combined shear–compression were very different to each other, as shown in Figure 19 and Figure 20. In Figure 19, the normal stress is reported vs. the crosshead displacement; the peak stress was reduced from 1.80 MPa in the configuration at a 0° application load to 1.04 MPa in the configuration at a 15° application load. The peak continued to decrease as the angle of the wedges increased (25°), with a value equal to 0.95 MPa. The plateau phase in the sample at 15° was very similar to the behavior at 0°, with a nearly constant trend. As the load application angle increased (>15°), the behavior of the honeycomb exhibited a decrease in the strength, which, in the direction out-of-the-plane, decreased significantly, as shown by the values of $\sigma_{m}^{*,n}$ equal to 0.98 for 0°, 0.93 for 15°, and 0.73 for 25°. Another interesting result was that the displacement corresponding to the start of densification in the combined compression (about 40 mm) was greater than that given in the normal compression (about 38 mm). This meant that the curve at an increasing force application angle became wider than that under uniaxial compression. Consequently, the honeycomb could be compressed to a decreasing thickness in the out-of-the-plane direction under combined stress rather than uniaxial stress. However, the displacement value corresponding to the beginning of densification decreased as the force application angle moderately decreased, varying from 0.75 mm at 15° to 0.73 mm at 25°. Figure 20 shows the shear stress/tangential displacement curve at different application force angles. The shear stress abruptly achieved a maximum value, and after that, it decreased with an increasing displacement, and then there was a final peak due to friction once all the cells collapsed. In general, as the force application angle increased, both the shear strength and the tangential displacement of the honeycomb increased up to densification. This was just the opposite of normal behavior, where, at an increasing load application angle, the strength decreases, as well the normal displacement up to densification. Regarding the total force curve (Figure 18), it was evident that an increase in the load application angle was connected to a shift of $\varepsilon_{d}^{*}$, but at the same time, a lowering of the curve and the plateau exhibited an increasing slope; this compensated for the displacement of $\varepsilon_{d}^{*},\;$ which therefore involved an increase in the absorbed energy, only in the case of a load application angle of 15° (equivalent to 0.72 $\frac{MJ}{m^{3}}$ vs. 0.67 $\frac{MJ}{m^{3}}$), as the plateau was almost not inclined and very similar to the curve with normal stress only. At 25°, the highest $\varepsilon_{d}^{*}$ and lower strength (due to the slope of the plateau with a decreasing σ_m_) led, overall, to a decrease in the absorbed energy, despite the increase in $\varepsilon_{d}^{*}$ (0.56 $\frac{MJ}{m^{3}}$). The energy associated with the shear deformation was not calculated due to the excessively irregular trend of the shear curve, but it was evident that the energy increased with an increasing load application angle, which justified the decrease in the total energy absorbed calculated at 25°. While for 15°, the shear energy was much lower compared to the total energy, the plateau was substantially unchanged compared to 0°, but at the same time, the deformation increased considerably and this justified the increase in the total absorbed energy calculated at 15°.

## 7. Conclusions and Future Outlook

An experimental investigation was carried out on aluminum hexagonal honeycombs under quasi-static combined compression–shear loadings in the out-of-the-plane- condition. A novel compression–shear loading device was designed and built using a couple of wooden wedges with different angles (15° and 25°). For each loading angle, the total stress vs. strain curve was obtained by the means of compression tests. In the loading condition with angles of load application of 15° and 25°, the total stress was decomposed in the normal stress and shear stress contributions. The cell rotation angle was calculated too.

From the analysis of the results, some main conclusions can be drawn, as reported in the following:

(1) Different deformation patterns were identified and correlated with the presence/absence of the shear component;
(2) The stress–strain curves were found to be significantly affected by the deformation pattern;
(3) The maximum peak stress, ascribable to the load instability, exhibited the maximum value for the 0° load application angle, while lower values could be found at 15° and 25°. In particular, the peak stress was reduced from 1.80 MPa at 0° to 1.04 MPa in the configuration at 15°, and 0.95 MPa at 25°;
(4) The plateau region was quite similar in the tests performed at 0° and 15°, while showing a sharp decrease in the stress at 25°, as evidenced by the $\sigma_{m}^{*,n}$, equal to 0.98 for 0°, 0.93 for 15°, and 0.73 for 25°;
(5) The stress–strain curves showed that the normal plateau stress decreased significantly with an increase in the load application angle;
(6) The displacement corresponding to the start of densification in the combined compression (about 40 mm) was greater than that given in the normal compression (about 38 mm); the honeycomb could be compressed to a decreasing thickness in the out-of-the-plane direction under combined stress rather than uniaxial stress;
(7) The shear stress abruptly achieved a maximum value, and after that, it decreased with an increasing displacement, and then there was a final peak due to friction once all the cells collapsed;
(8) The absorbed energy increased from 0.67 $\frac{MJ}{m^{3}}$ at 0° to 0.72 $\frac{MJ}{m^{3}}$ at 15° and decreased again to 0.56 $\frac{MJ}{m^{3}}$ at 25°, due to the contribution of higher displacement, but at the same time, lower stress;
(9) The presented results are in good agreement with other research [19,20], in which normal strength decreases while shear strength increases with the loading angle increasing, changing the deformation mode from the progressive folding mode to the global rotation mode;
(10) The application of a shear stress component during operation, in addition to the normal compression, may result in a premature failure of the component, with a lower stress than expected.

The experimental set-up for carrying out tests at angles increasing up to 45° is being acquired.

## Figures and Tables

**Table 1:** Honeycomb technical specifications, as declared by the supplier.

Alveolar diameter f (mm)	9
Density (kg/m^3^)	30–40
Strength at stabilized compression (MPa)	1.4–1.9
Young modulus (GPa)	70
Shear modulus (GPa)	20

**Table 2:** List of measured engineering properties.

Maximum normal stress after linear stage (Peak stress)	$\sigma_{pk}^{*}$
Average normal stress (Plateau stress)	$\sigma_{m}^{*}$
Energy absorption efficiency	$\eta_{max}^{*}$
Strain (e*) corresponding to maximum energy efficiency $\eta_{max}^{*}$	$\varepsilon_{d}^{*}$
Absorbed energy	$U^{*}$
Maximum shear stress	$\tau_{pk}^{*}$

**Table 3:** Main results from tests performed at 0°.

Sample #	$\sigma_{pk}^{*}[MPa]$	$\sigma_{m}^{*}[MPa]$	$\eta_{max}^{*}$	${ɛ}_{d}^{*}$	U*[MJm3]
2	1.80	0.98	0.65	0.68	0.67
3	1.96	0.93	0.62	0.65	0.60

**Table 4:** Main results from tests performed at 15°.

Sample #	$\sigma_{pk}^{*}[MPa]$	$\sigma_{m}^{*}[MPa]$	$\eta_{max}^{*}$	${ɛ}_{d}^{*}$	U*[MJm3]
1	1.07	0.94	0.79	0.76	0.71
4	1.03	0.93	0.72	0.77	0.72

**Table 5:** Main results of tests performed at 25°.

Sample #	$\sigma_{pk}^{*}[MPa]$	$\sigma_{m}^{*}[MPa]$	$\eta_{max}^{*}$	${ɛ}_{d}^{*}$	U*[MJm3]
5	1.01	0.76	0.87	0.77	0.59
6	0.93	0.69	0.85	0.81	0.56

**Table 6:** Stress–total strain values for tests performed at 0°, 15°, and 25°.

Sample 0°	$\sigma_{pk}^{*}[MPa]$	$\sigma_{m}^{*}[MPa]$	$\eta_{max}^{*}$	${ɛ}_{d}^{*}$	U*[MJm3]
2	1.80	0.98	0.65	0.68	0.67
3	1.96	0.93	0.62	0.65	0.60
**Sample 15°**	$\sigma_{pk}^{*}[MPa]$	$\sigma_{m}^{*}[MPa]$	$\eta_{max}^{*}$	${ɛ}_{d}^{*}$	U*[MJm3]
1	1.07	0.94	0.79	0.76	0.71
4	1.03	0.93	0.72	0.77	0.72
**Sample 25°**	$\sigma_{pk}^{*}[MPa]$	$\sigma_{m}^{*}[MPa]$	$\eta_{max}^{*}$	${ɛ}_{d}^{*}$	U*[MJm3]
5	1.01	0.76	0.87	0.77	0.59
6	0.93	0.69	0.85	0.81	0.56

## Data Availability

No new data were created.
